# Draft Genome Sequence of an Anaerobic and Extremophilic Bacterium, *Caldanaerobacter yonseiensis*, Isolated from a Geothermal Hot Stream

**DOI:** 10.1128/genomeA.00923-13

**Published:** 2013-11-07

**Authors:** Sang-Jae Lee, Yong-Jik Lee, Gun-Seok Park, Byoung-Chan Kim, Sang Jun Lee, Jae-Ho Shin, Dong-Woo Lee

**Affiliations:** School of Applied Biosciences, Kyungpook National University, Daegu, South Koreaa; Biological Resource Center, Korean Collection for Type Culture (KCTC), Korea Research Institute of Bioscience and Biotechnology (KRIBB), Daejeon, South Koreab; Infection and Immunity Research Center, Korea Research Institute of Bioscience and Biotechnology (KRIBB), Daejeon, South Koreac

## Abstract

*Caldanaerobacter yonseiensis* is a strictly anaerobic, thermophilic, spore-forming bacterium, which was isolated from a geothermal hot stream in Indonesia. This bacterium utilizes xylose and produces a variety of proteases. Here, we report the draft genome sequence of *C. yonseiensis*, which reveals insights into the pentose phosphate pathway and protein degradation metabolism in thermophilic microorganisms.

## GENOME ANNOUNCEMENT

*Caldanaerobacter yonseiensis* was isolated from a geothermal hot stream at Sileri, Java Island, in Indonesia (1). Previously, this strain was classified as a new species of the genus *Thermoanaerobacter*, designated *Thermoanaerobacter yonseiensis*, but reassigned as a novel genus and species, *Caldanaerobacter yonseiensis* (2). This strain is a strictly anaerobic, extremely thermophilic, spore-forming, and xylose-utilizing bacterium. *C. yonseiensis* can grow in the temperature range of 50 to 85°C, with an optimum at 75°C, and a pH range for growth of 4.5 to 9.0, with an optimum at pH 6.5 (1). These properties indicated that *C. yonseiensis* might be a good source for the isolation of thermostable and acidophilic d-xylose isomerase, which is suitable for the production of high fructose corn syrup in the food industry (3–5). Moreover, *C. yonseiensis* produces a novel subtilisin-like protease, thermicin, that showed maximum proteolytic activity at 92.5°C and pH 9.0, indicating that this enzyme will be applicable for the hydrolysis of collagens at elevated temperatures without contamination (6). Thus, *C. yonseiensis* is of great interest as a potential industrial microorganism, which led us to sequence the whole genome of this microorganism.

The genome of *C. yonseiensis* was sequenced via the Ion Torrent personal genome machine (PGM) sequencer system using a 316 D sequencing chip (7). The sequence was assembled using MIRA 3.4.0. The assembled genome consists of 102 contigs (>500 bp), with a genome size of 2,700,546 bp at 34.96-fold coverage, with a G+C content of 36.6%. The assembled contigs were submitted to the RAST annotation server (http://rast.nmpdr.org/) for subsystem classification and functional annotation. There were a total of 2,905 predicted protein-coding sequences (CDS), with 49% assigned to recognizable functional genes. While the 16S rRNA gene sequencing of *C. yonseiensis* showed the similarity of this bacterium to *C. tengcongensis* strain MB4 (99% sequence identity) and *C. keratinophilus* strain 2KXI (99% sequence identity), RAST analysis suggested that *C. tengcongensis* MB4 was actually the closet neighbor in terms of sequence similarity.

Consequently, we confirmed 13 genes from the draft sequence that encode proteins related to xylose utilization, including two xylose isomerase-coding genes that are involved in xylose metabolism in the *C. yonseiensis* genome. Also identified were genes that encode glucose-6-phosphate 1-dehydrogenase and 6-phosphogluconate dehydrogenase, which are among the key enzymes for the generation of reducing power (NADPH) in the pentose phosphate pathway. Another feature of *C. yonseiensis* is the ability to hydrolyze glycine- and proline-rich collagens (6). Based on our data, a gene encoding a subtilicin-like serine protease, designated thermicin, was identified, together with another four genes encoding serine proteases. Moreover, four genes encoding cysteine proteases and 27 genes encoding a variety of proteases were identified. Given these results, the draft genome sequence of *C. yonseiensis* will not only provide insights into the metabolism of monomeric and polymeric carbohydrates under extremely thermophilic conditions, but it will also encourage further study of a variety of proteases potentially applicable for their use in detergents and in the leather and textile industries.

### Nucleotide sequence accession numbers.

This whole-genome shotgun project has been deposited at DDBJ/EMBL/GenBank under the accession number AXDC00000000. The version described in this paper is the first version, AXDC01000000.

## References

[1] Kim BC Grote R Lee DW Antranikian G Pyun YR 2001 *Thermoanaerobacter yonseiensis* sp. nov., a novel extremely thermophilic, xylose-utilizing bacterium that grows at up to 85 degrees C. Int. J. Syst. Evol. Microbiol. 51:1539–1548 11491356 10.1099/00207713-51-4-1539

[2] Fardeau ML Bonilla Salinas M L’Haridon S Jeanthon C Verhé F Cayol JL Patel BK Garcia JL Ollivier B 2004 Isolation from oil reservoirs of novel thermophilic anaerobes phylogenetically related to *Thermoanaerobacter subterraneus*: reassignment of *T. subterraneus*, *Thermoanaerobacter yonseiensis*, *Thermoanaerobacter tengcongensis* and *Carboxydibrachium pacificum* to *Caldanaerobacter subterraneus* gen. nov., sp. nov., comb. nov. as four novel subspecies. Int. J. Syst. Evol. Microbiol. 54:467–474 15023962 10.1099/ijs.0.02711-0

[3] Dekker K Yamagata H Sakaguchi K Udaka S 1991 Xylose (glucose) isomerase gene from the thermophile *Clostridium thermohydrosulfuricum*; cloning, sequencing, and expression in *Escherichia coli*. Agric. Biol. Chem. 55:221–227 1368665

[4] Dekker K Yamagata H Sakaguchi K Udaka S 1991 Xylose (glucose) isomerase gene from the thermophile *Thermus thermophilus*: cloning, sequencing, and comparison with other thermostable xylose isomerases. J. Bacteriol. 173:3078–3083 2022613 10.1128/jb.173.10.3078-3083.1991 PMC207900

[5] Liu SY Wiegel J Gherardini FC 1996 Purification and cloning of a thermostable xylose (glucose) isomerase with an acidic pH optimum from *Thermoanaerobacterium* strain JW/SL-YS 489. J. Bacteriol. 178:5938–5945 8830690 10.1128/jb.178.20.5938-5945.1996 PMC178450

[6] Jang HJ Kim BC Pyun YR Kim YS 2002 A novel subtilisin-like serine protease from *Thermoanaerobacter yonseiensis* KB-1: its cloning, expression, and biochemical properties. Extremophiles 6:233–243 12072959 10.1007/s00792-001-0248-1

[7] Rothberg JM Hinz W Rearick TM Schultz J Mileski W Davey M Leamon JH Johnson K Milgrew MJ Edwards M Hoon J Simons JF Marran D Myers JW Davidson JF Branting A Nobile JR Puc BP Light D Clark TA Huber M Branciforte JT Stoner IB Cawley SE Lyons M Fu Y Homer N Sedova M Miao X Reed B Sabina J Feierstein E Schorn M Alanjary M Dimalanta E Dressman D Kasinskas R Sokolsky T Fidanza JA Namsaraev E McKernan KJ Williams A Roth GT Bustillo J 2011 An integrated semiconductor device enabling non-optical genome sequencing. Nature 475:348–352 21776081 10.1038/nature10242

